# A Comparison of Neutral and Immune Genetic Variation in Atlantic Salmon, *Salmo salar* L. in Chilean Aquaculture Facilities

**DOI:** 10.1371/journal.pone.0099358

**Published:** 2014-06-11

**Authors:** David S. Portnoy, Christopher M. Hollenbeck, R. Rodrigo Vidal, John R. Gold

**Affiliations:** 1 Department of Life Sciences, Texas A&M University, Corpus Christi, Texas, United States of America; 2 Marine Genomics Laboratory Harte Research Institute, Texas A&M University, Corpus Christi, Texas, United States of America; 3 Laboratory of Molecular Ecology, Genomics, and Evolutionary Studies, Department of Biology, University of Santiago de Chile, Santiago, Chile; INRA, France

## Abstract

Genetic diversity was assessed in samples of cultured Atlantic salmon, *Salmo salar* L., obtained from facilities in Chile between 2005 and 2010, a period of time during which the infectious pathogens Infectious Salmon Anemia (ISA) virus, *Caligus rogercresseyi* (sea lice), and *Piscirickettsia salmonis* (salmon rickettsial syndrome) were common. Two panels of microsatellite markers were utilized: one with microsatellites with no known gene associations (neutral) and one featuring microsatellites linked to putative immune-related genes (immune-related). Allelic richness and gene diversity across samples were significantly greater in neutral loci as compared to immune-related loci. Both diversity measures were homogeneous among samples for immune-related loci and heterogeneous among samples for neutral loci. Immune-related loci were identified as *F_ST_* outliers in pairwise comparisons of samples at a 10-fold higher frequency than neutral loci. These results indicate that neutral and immune-related portions of the Atlantic salmon genome may have differed in response to the gauntlet of pathogens and that monitoring of specific, well characterized immune-related loci as well as neutral loci in cultured species could be useful when disease control and prevention is a goal.

## Introduction

Genetic assessment has become a valuable component of many aquaculture programs. Keeping track of parentage, marker-assisted selection, and monitoring standing genetic variability of brood stocks are among the more important applications [1]. Selectively neutral, single-copy markers such as microsatellites have been used widely because their design and implementation are fairly straightforward, allowing comparisons between wild and captive populations [2]–[4]. In addition, measures of variability obtained with large panels of microsatellites often are taken as a proxy for population-wide fitness [5]–[6]. The strength of the correlation between neutral genetic variation and aspects of quantitative genetic variation associated with fitness, however, is controversial [7]–[8]. In settings with differential selection pressures, levels of variation may differ between parts of the genome which are selectively neutral and those associated with adaptive fitness [9]. This could be a concern in aquaculture facilities where unintended selective regimes caused by pathogens and parasites or unanticipated mass mortality may alter adaptive parts of the genome yet leave neutral parts unaffected [10].

Disease management in the culture of Atlantic salmon, *Salmo salar* L., has become a particularly important issue worldwide as a number of potentially devastating pathogens, sometimes in concert, have impacted the global industry. Examples include infectious pancreatic necrosis virus or IPNV [11], salmonid alphavirus or SAV [12], infectious salmon anemia virus or ISA [13]–[14], and a variety of parasites [15]. A recent ISA epizootic in Chile, which began in 2007, was particularly devastating to the Chilean salmon industry, which by 2005 had grown to be the second largest producer of Atlantic salmon in the world [16]. While initial mortalities attributable to the virus were documented in 2007, the virus likely was present as many as eleven years prior to its detection [17]. To complicate matters further, sea lice (*Caligus rogercresseyi*) were prevalent across the Chilean aquaculture industry [18] and may have increased susceptibility of fish to ISA and facilitated spread of the virus [19]–[20]. In addition, salmon rickettsial syndrome, caused by the intracellular bacteria *Piscirickettsia salmonis*, had been an issue in Chilean aquaculture facilities since the late 1980s [21]. The net result of these pathogens across the industry in Chile was a reduction in production of approximately two-thirds due to direct mortality and/or culling [22]. Because of the exogenous origin of the ISA virus [17], part of the recovery plan banned the importation of salmon ova [23]; consequently, present-day brood stocks in Chile are an amalgam of shared individuals that survived the epizootic. By 2010, when the industry began to recover, there was a loss of ∼30% of export value and a ∼50% reduction of the associated work force [23].

We assessed genetic diversity within and among samples from seven different farms in Chile. The samples were part of a tissue bio-bank maintained at the University de Santiago de Chile and procured between 2005 and 2010. Two panels of microsatellite markers were utilized: one featuring microsatellites not known to be associated with functional genes (neutral loci), and the other featuring microsatellites known to be linked to putative functional immune genes (immune-related loci). The primary goal of this study was to assess whether patterns of genetic diversity within and among samples obtained from selectively neutral loci were consistent with patterns of diversity obtained from loci which potentially could be under selection.

## Materials and Methods

Tissues from a total of 109 Atlantic salmon were obtained by R. Vidal from seven aquaculture facilities in Chile. Samples were obtained from live fish by a third-party diagnostic laboratory that screens aquaculture farms for infectious agents (Diagnotec S.A.) We obtained tissues that had been stored at the University de Santiago de Chile. There is no official animal care protocol in Chile. However, salmon aquaculture is an enormous industry (second largest salmon aquaculture industry in the world) and aquaculture farms are tightly regulated due to past problems with widespread disease. Therefore all farms had necessary permits. Because of commercial confidentiality issues, individual companies and localities of each facility are not named. Samples from each individual source farm were labeled A – G. Samples A – C were obtained in 2010 when recovery was underway; whereas samples D – G were obtained between 2005 and 2007 during a period of high disease-related mortality. Sample sizes ranged from 10 (sample G) to 30 (sample A). Tissues were taken directly from brood stock and preserved in 20% DMSO buffer [24]. DNA was extracted with a modified Chelex extraction protocol [25]. After centrifugation for two minutes at 13,000×g, the resulting supernatant was used as template for PCR amplification.

Individuals were genotyped at 30 microsatellite loci; 15 of the 30 loci assayed were reported in previous studies to behave as selectively neutral [26], [27], [28], [29] (hereafter, neutral), while the other 15 were reported to be tightly linked to genes relating to immune function in salmon (hereafter, immune-related). Information for each microsatellite, including the original reference, is given in Table 1. Each microsatellite was amplified using either a fluorescently labeled forward primer or using the ‘tail-labeled’ methodology reported in Karlsson *et al.* [30]. In both cases, one of three fluorescent dyes, 6-Fam, Ned, or Hex (Dye Set D, Applied Biosystems), was used. Polymerase chain reaction (PCR) amplification, using fluorescently labeled primers, was carried out in 10 µL reactions containing: 1x reaction buffer, 2 mM MgCl_2_, 0.25 mM of each dNTP, 5 picomoles of each primer, 0.05 U/µL of *Taq* polymerase, and 50–150 nanomoles of DNA template. Reaction conditions consisted of an initial denaturation at 95°C for 3 min, followed by 38 cycles of 95°C for 30 sec, 58°C for 45 sec, and 72°C for 60 sec, and followed by a final extension at 72°C for 10 min. PCR amplification using the tail-labeled methodology was carried out in 10 µL reactions containing: 1x reaction buffer, 2 mM MgCl_2_, 0.25 mM of each dNTP, 5 picomoles of the tail and reverse primers, 0.5 picomoles of forward primer, 0.05 U/µL of *Taq* polymerase, and 50–150 nanomoles of DNA template. Reaction conditions consisted of an initial denaturation at 95°C for 15 min, followed by 14 cycles of 95°C for 30 sec, 59°C for 90 sec, and 72°C for 60 sec and 24 cycles of 95°C for 30 sec, 52°C for 90 sec, and 72°C for 60 sec, followed by a final extension at 60°C for 30 min. Amplicons were electrophoresed and visualized on 6% polyacrylamide gels, using an ABI 377 Automated Sequencer (Applied Biosystems). Allele calling was performed manually, using Genotyper 2.5 (Perkin Elmer) and Genescan 3.1.2 (Applied Biosystems).

**Table 1:** Panels of neutral loci and immune-related loci used to screen samples of Atlantic salmon, *Salmo salar*, from the salmon industry in Chile.

Locus Type	Motif^a^	Range^b^	T_A_ c	Label^d^	Primers^e^	Reference^f^
**Neutral**						
*SSsp*1605	(GATA)_11_	214–252	58	Fam	F: CGCAATGGAAGTCAGTGGACTGGR: CTGATTTAGCTTTTTAGTGCCCAATGC	[50]
*SSsp*2201	(GATA)_34_	251–335	58	Fam	F: TTTAGATGGTGGGATACTGGGAGGCR: CGGGAGCCCCATAACCCTACTAATAAC	[50]
*SSsp*2210	(GTTA)_11_	112–168	58	Fam	F: AAGTATTCATGCACACACATTCACTGCR: CAAGACCCTTTTTCCAATGGGATTC	[50]
*SSsp*2213	(GTTA)_22_	151–215	58	Hex	F: ATGTGGAGGTCAACTAACCAGCGTGR: CATCAATCACAGAGTGAGGCACTCG	[50]
*SSsp*2215	(GTTA)_14_	107–175	58	Ned	F: ACTAGCCAGGTGTCCTGCCGGTCR: AGGGTCAGTCAGTCACACCATGCAC	[50]
*SSsp*2216	(GTTA_)25_	201–257	58	Ned	F: GGCCCAGACAGATAAACAAACACGCR: GCCAACAGCAGCATCTACACCCAG	[50]
*SSsp*G7	(GTTA)_18_	121–215	58	Hex	F: CTTGGTCCCGTTCTTACGACAACCR: TGCACGCTGCTTGGTCCTTG	[50]
*SSa*85	(GT)_14_	106–186	58	Ned	F: AGGTGGGTCCTCCAAGCTACR: ACCCGCTCCTCACTTAATC	[51]
*SSa*197	(GT)_5_C(TG)_4_TC(TG)_3_A(GTGA)_15_	162–260	58	Fam	F: GGGTTGAGTAGGGAGGCTTGR: TGGCAGGGATTTGACATAAC	[51]
*SSa*171	(TGTA)_14_(TG)_7_	198–252	58	Fam	F: TTATTATCCAAAGGGGTCAAAAR: GAGGTCGCTGGGGTTTACTAT	[51]
*SSa*202	(CA)_3_(CTCA)_17_	237–371	58	Ned	F: CTTGGAATATCTAGAATATGGCR: TTCATGTGTTAATGTTGCGTG	[51]
*SSa*14	(TC)_10_N_15_(TC)_3_N_2_(AC)_12_(TC)_3_N_5_(CA)_4_	161–165	NA	Tail	F: CCTTTTGACAGATTTAGGATTTCR: CAAACCAAACATACCTAAAGCC	[52]
*SS*O*SL85*	(GT)_22_	201–241	NA	Tail	F: TGTGGATTTTTGTATTATGTTAR: ATACATTTCCTCCTCATTCAGT	[53]
*SS*OSL311	(TG)_38_	139–190	NA	Tail	F: TAGATAATGGAGGAACTGCATTCTR: CATGCTTCATAAGAAAAAGATTGT	[53]
*SS*OS*L438*	(AC)_26_AT(AC)_8_	132–174	NA	Tail	F: GACAACACACAACCAAGGCACR: TTATGCTAGGTCTTTATGCATTGT	[53]
**Locus Type**	**Motif** a	**Range** b	**T_A_** c	**Label** d	**Primers** e	**Reference** f
**Immune-related**						
*Ssa*IR001TKU	(AG)_12_	113–115	NA	Tail	F: AAGAGCGAGAGAGAAGGATGGR: GTTTCACAGAATCAACAGTCAGCAA	[47]
*Ssa*IR002TKU	(AG)_10_	241–249	NA	Tail	F: GGGTACAAGCAGGGGTCTTAR: GTTTAAGAGTGGACCGACAACAAT	[47]
*SsaIR003TKU*	(GT)_13_	146–163	NA	Tail	F: TGTTCTGCAGGTCAGAAGTGAR: GTTTGAGTGGGAGGAAGGGGAGTA	[47]
*Ssa*IR004TKU	(AG)_10_	313–321	NA	Tail	F: AGCTATTTCCAAGGCGTTCAR: GTTTCACCACTCAGGAGAGCATGA	[47]
*SsaIR005TKU*	(CTT)_7_	371–379	NA	Tail	F: CGACGACTTTTTCATCTGTCTTR: GTTTGGACAACACATCTCATTCCAA	[47]
*SsaIR007TKU*	(AG)_9_	183–191	NA	Tail	F: GCAATGCTGCCATCTAGTGAR: GTTTCAAGGAAAGCCTACAAAAAGC	[47]
*SsaIR010TKU*	(AG)_10_	192–198	NA	Tail	F: CAACGACACCATACCAACCAR: GTTTAACCCCTTCCAAGTTCCATC	[47]
*Ssa*IR011TKU	(ATC)_8_	364–376	NA	Tail	F: CCAGCCAACTACGACAACTGR: GTTTGTGGTTATTTTTGGGGTGA	[47]
*Ssa*IR012TKU	(AATC)_14_	162–222	NA	Tail	F: GAGTCCCCTTTGGCCTCTCR: GTTTAAACACAGTAAGCCCATCTATTG	[47]
*Ssa*IR014TKU	(ATC)_6_	395–407	NA	Tail	F: CTGAGGTGGTGGCACAGCR: GTTTATTGTTTGGTTCTTACAGCAGGA	[47]
*Ssa*IR016TKU	(AT)_9_	317–327	NA	Tail	F: CCAAAAATGTCCCATTCACCR: GTTTGTGTGCCACTCAGAATTG	[47]
*BG9*34281	(TCTG)_14_	215–277	NA	Tail	F: ACTGCTTCTCCCCTGCTACAR: GTTTGCGAACCACACATATACCAC	[46]
*CA*769358	(AC)_22_	133–173	NA	Tail	F: TGACGCCATATGCAAAGAGAR: GTTTCCTTTGTCTGCAAAACGTGA	[46]
UBA	(CA)17	330–364±	NA	Tail	F: GGAGAGCTGCCCAGATGACTTR: CAATTACCACAAGCCCGCTC	[44]
MHC_II	GTTATTAAAT	232–292	NA	Tail	F: GATGGCAAAGAGGAAAGTGAGR: TTGTTATGCTCTACCTCTGAA	[45]

a Motif indicates repeat motif.

b Range refers to alleles thus far uncovered (may include the 21 bp 50-tail-sequence).

c T_A_ is optimized annealing temperature. For loci amplified with the tail protocol, a step-down protocol was used so annealing temperature is not applicable (NA).

d Label refers to dye (6-Fam, Ned, or Hex) used to label forward primer. Tail refers to loci that were amplified using the tail protocol.

e Primer sequences are forward (F and reverse (R).

f Citations describing putative function.

Conformance to expectations of Hardy-Weinberg equilibrium (HWE) was evaluated for each microsatellite in each sample, using Genepop v.4.0 [31]–[32]; significance was assessed at the 0.05 level, using exact tests, with 1,000 batches and 10,000 iterations per batch. Sequential Bonferroni adjustment [33] was used to correct for multiple testing. Allelic richness (the expected number of alleles standardized to the smallest sample size using rarefaction) and unbiased gene diversity (expected heterozygosity) were estimated for each microsatellite in each sample, using Fstat v.2.9.3.2 [34]–[35]. Wilcoxon signed-rank tests, implemented in Systat 8.0 (SPSS Inc.), were used to test for homogeneity in allelic richness and gene diversity between marker panels (neutral vs. immune-related). Friedman rank tests, as implemented in Systat, were used to test homogeneity of allelic richness and gene diversity across samples for both neutral and immune-related loci separately. Finally, linear regression was used to test for a correlation between diversity estimates made with both marker panels (neutral and immune-related) across samples.

Lositan [36] was used to screen for *F_ST_* outliers (candidate loci under selection) by comparing observed mean pairwise *F_ST_* values at each microsatellite between pairs of samples; mean *F_ST_* values were corrected for locus-specific gene diversity (expected heterozygosity), against a 95% and 99% confidence interval of *F_ST_* values (corrected for diversity) generated by simulation. Simulations were implemented using the stepwise mutational model with 60,000 steps and an initial simulation of 60,000 steps run to estimate mean neutral *F_st_*.

## Results

**Figure 1:**
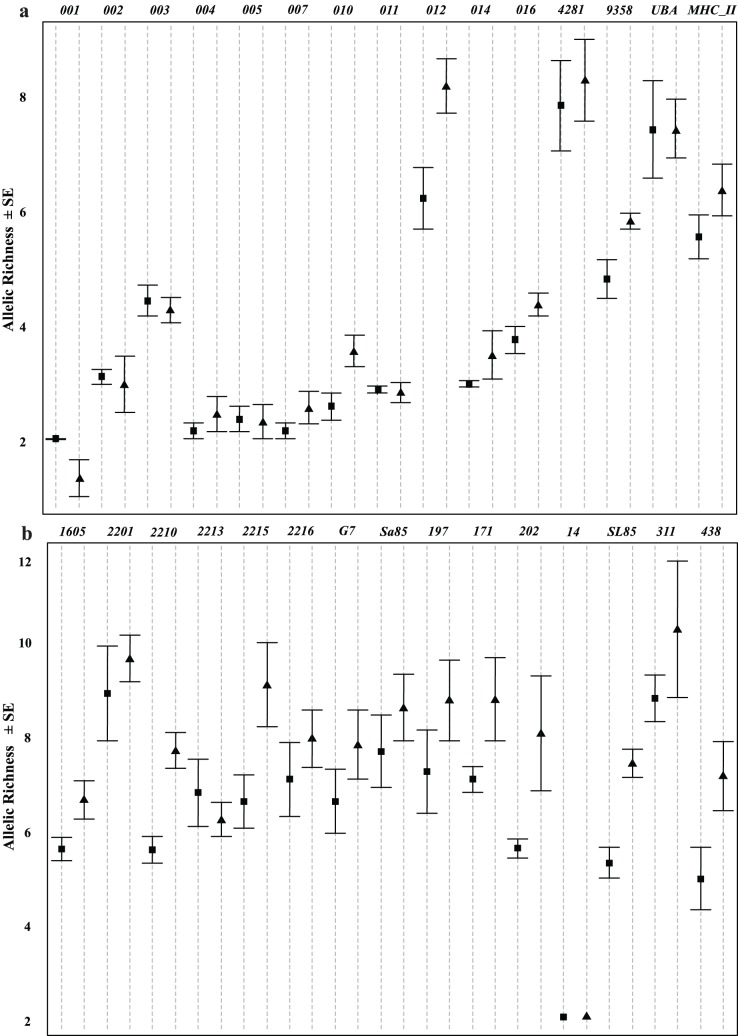
Comparisons of mean allelic richness ± standard error for neutral loci (a) and immune loci (b) for samples taken during the epizootic (square) and sample taken during recovery (triangle). Locus names (see Table 1) are abbreviated. Figure created using R v.2.1.3.1 [57], using dotplots.errors [58].

Genotypes at all microsatellites assayed are in Data Set S1. Summary statistics for all microsatellites in each sample are presented in Table S1. Genotypes at all microsatellites conformed to the expectations of HWE, following correction for multiple tests. Mean allelic richness assayed per sample (± S.E.) for neutral loci ranged from 5.68±0.34 (Sample F) to 8.34±0.66 (Sample B) and for immune-related loci ranged from 3.50±0.35 (Sample G) to 4.54±0.62 (Sample A). Mean gene diversity assayed per sample (± S.E.) for neutral loci ranged from 0.746±0.043 (Sample G) to 0.840±0.044 (Sample B) and from 0.516±0.073 (Sample B) to 0.602±0.058 (Sample A) for immune-related loci. Wilcoxon signed-rank tests revealed that both allelic richness (*Z*  = 2.366; *P*  = 0.018) and gene diversity (*Z*  = 2.366; *P*  = 0.018) in neutral loci were significantly greater than in immune-related loci. Friedman rank tests revealed different patterns of genetic variability within marker types. Both allelic richness (*Q*  = 41.461, *P*<0.001) and gene diversity (*Q*  = 19.143, *P*  = 0.004) of neutral loci were heterogeneous across samples; whereas the two parameters were homogeneous across samples when immune-related loci were considered (*Q*  = 9.696, *P*  = 0.138– and *Q*  = 7.309, *P*  = 0.293, respectively, Fig. 1.). Post-hoc, sign-rank analysis of variance (Anova), using just neutral loci, revealed significant differences in both allelic richness (*F*  = 36.854; *P*<0.001) and gene diversity (*F*  = 17.749, *P*<0.001) between samples taken between 2005–2007 and those taken in 2010 with a higher mean diversity for both measures in the samples from 2010 (allelic richness –13.89±1.31 vs. 11.84±1.10; gene diversity –0.848±0.030 vs. 0.807±0.031, Fig. 1). No significant correlation was found across samples between estimates of diversity based on the neutral loci and those based on immune-related loci (allelic richness: *F*_ [1,5]_  = 2.471, *P*  = 0.177, gene diversity: *F*_ [1,5]_ *= *0.155, *P*  = 0.710).

**Figure 2:**
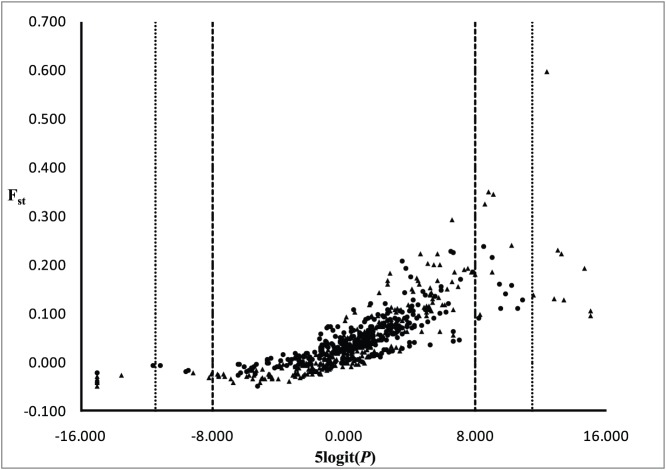
Results of outlier tests: ordinate (pairwise F_ST_ values); abscissa (logit transformed probability values). Heavy dashed lines indicate significance at α  = 0.05; light dotted lines indicate significance at α  = 0.01. Circles are neutral loci; triangles are immune-related loci.

A total of 43 (6.8%) outlier *F_st_* values (at α  = 0.05) were detected among 630 pairwise comparisons of samples at each of the 30 microsatellites markers (Fig. 2). Of these, 13 involved neutral loci; the remainder (30 comparisons) involved immune-related loci. A total of 22 (3.5%) *F_st_* outliers were detected at α  = 0.01 (Fig. 2); of these, two involved one of the 15 neutral loci assayed, while the remainder involved nine of the 15 immune-related loci assayed (Fig. 2). Of the 20 *F_ST_* outliers involving the nine immune-related loci, 10 had *F_ST_* values that exceeded neutral expectations, and nine of these 10 involved comparisons between samples taken between 2005 and 2007 and samples taken during 2010. In theory, *F_ST_* values that significantly exceed neutral expectations may reflect directional or diversifying selection [37]–[38]. The immune-related loci linked to genes that may have been under directional or diversifying selection were *SSa*I001TKU, *SSa*I004TKU, and *Ssa*I012TKU, and CA769358; three of which have been inferred in other species to be involved in response to inflammation and infection by hemorrhagic septicemia virus (Table 2). It is also worth noting that at the less stringent (α  = 0.05) level, all but one of the 17 *F_ST_* values that exceeded neutral expectations involved one of these same four immune-related loci.

**Table 2:** Immune microsatellites that were significant positive outliers, at α  = 0.01, in pairwise comparisons of samples.

Locus	Panel	Coding Genes	Function	Citation
*SSa*I001TKU	Immune	–	–	–
*SSa*I004TKU	Immune	Interleukin-1 β	Mediator of inflammatory response	[54]
*Ssa*I012TKU	Immune	Neutrophil chemotactic factor	Involved in inflammatory reactions	[55]
CA769358	Immune	VHSV-induced protein	Response to hemorrhagic septicemia virus	[56]

Coding genes, and their putative function, to which immune-related markers are potentially linked are indicated.

## Discussion

We examined patterns of variability in selectively neutral microsatellites and in microsatellites closely linked to immune-related genes within and among samples of cultured Atlantic salmon exposed to a number of pathogens. Part of our goal was to assess whether neutral genetic diversity was a suitable proxy for adaptive genetic diversity. Tests of homogeneity in allelic richness and gene diversity between the two genetic marker types across all samples revealed significantly greater allelic richness and gene diversity in neutral loci. This finding strongly indicates that the selectively neutral loci employed were not appropriate proxies for the immune-related loci, and moreover, that there may be selective constraints on the diversity of immune-related loci, given that strong selection against functional allelic variants can lead to reduced variation at closely linked genetic markers [39]. In addition, because addition or subtraction of repetitive elements can alter gene function, there may be selective constraints on polymorphism of microsatellites proximal to coding regions [40]–[41]. Consistent with this, the average range of allele size (correlated with number of alleles) within loci differed significantly between neutral and immune-related loci (*t*_ [14]_ = −3.60, *P*  = 0.003), with a higher mean of 64.3 bp for neutral loci versus 23.3 bp for immune-related loci. Levels of variation in neutral loci, measured as both allelic richness and gene diversity, also were significantly heterogeneous among samples, with greater levels of diversity present in samples taken in 2010 (as the industry recovered). This is perhaps due to restocking with a mix of individuals from other Chilean aquaculture farms which may not have had high levels of diseases resulting in crosses of relatively outbred individuals. Levels of variation in immune-related loci, however, were homogeneous and there was no significant correlation across samples in levels of variation (allelic richness and gene diversity) of the two marker types. This, too, indicates that neutral loci are not suitable proxies for immune-related loci, failing to capture both patterns and levels of immune variation.

**Figure 3:**
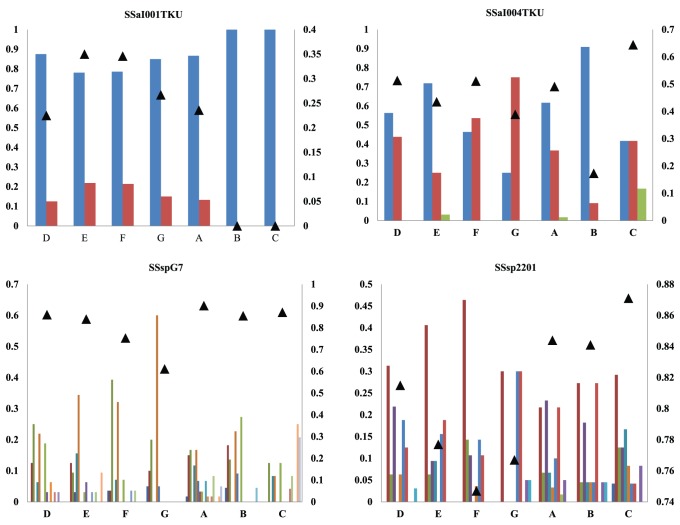
Allele frequency distributions (bars, left axis) and unbiased gene diversity (triangles, right axis) for two immune loci that were significant positive outliers and for two neutral loci.

The two genetic marker types differed substantially at both α  = 0.05 and α  = 0.01 in the number of *F_ST_* outliers detected in pairwise comparisons of samples, with far fewer pairwise *F_ST_* outliers involving neutral loci than involving immune-related loci. At both statistical levels, *F_ST_* outliers that exceeded neutral expectations primarily involved immune-related loci, and most of these involved pairwise comparisons between samples taken between 2005 and 2007 and samples taken during 2010. In general, *F_ST_* values that significantly exceed neutral expectations are hypothesized to reflect directional or diversifying selection [37]–[38], suggesting that allele-frequency changes at immune-related loci might have occurred in response to the gauntlet of pathogens present in Chilean aquaculture farms in the mid-to-late 2000s and that these changes were independent of overall levels of diversity. Consistent with this, diversity in outlier loci is higher for two of the loci (*Ssa*I012TKU and CA769358), lower for another (*SSa*I001TKU, Fig. 3a), and equivocal for the fourth (SsaI004TKU, Fig. 3b) when samples taken between 2005 and 2007 are compared with samples taken in 2010. By contrast, neutral markers revealed a pattern of increased diversity when samples taken between 2005 and 2007 are compared with samples taken in 2010 (Fig. 3c–d). Because the original purpose in obtaining samples at various aquaculture farms was to have a tissue database of salmon brood stock in Chile, no tissue samples were available from the same facilities taken at different time periods. In addition, confidentiality issues precluded acquisition of further samples. Consequently, while we are able to document change in the frequency of several alleles at the four, immune-related loci (*SSa*I001TKU, *SSa*I004TKU, and *Ssa*I012, and CA769358) where *F_ST_* values significantly exceeded neutral expectations, we cannot unequivocally demonstrate that the changes occurred at the same aquaculture facility. However, the putative functions of three of the four principle immune-related loci involve responses to inflammation and/or to hemorrhagic viral infection, and two of the loci (*SSa*I004TKU and CA769358) have been detected as *F_ST_* outliers and taken as evidence of directional selection in wild populations of Atlantic salmon [29]. Consistent with this is the finding that only two *F_ST_* outliers (*SSsp*1605 and *SSsp*2210) involved neutral loci, and in both cases estimated *F_ST_* values were less than expected under a model of selective neutrality.

While we cannot unequivocally associate the allele changes observed at the immune-related loci with the diseases present; the immune-related loci identified in this study may represent a good starting point for future genomics research. Given that sequencing of the Atlantic salmon genome is near completion [42]–[43] and that expressed-sequence-tags (ESTs) used to design PCR primers for the immune-related loci in our study are available [44]–[47], it should be straightforward to identify suitable polymorphic markers (e.g., SNPs) to complement already ongoing genomics and other studies [48]–[49] searching for genes conferring resistance to pathogens.

## Conclusions

This study demonstrates that patterns of variation in assumed selectively neutral genetic loci differ substantially from patterns of variation in immune-related genetic loci in Atlantic salmon exposed to a suite of pathogens. Both levels of genetic variation (greater in neutral loci) and number of pairwise comparisons, where *F_ST_* values exceeded neutral expectations (greater in immune-related loci), are consistent with the hypothesis that allelic variation at the immune-related loci may have been directly or indirectly affected by presence of multiple pathogens. Putative immune loci affected by the infection include genes hypothesized to be involved in response to inflammation and infection by a hemorrhagic septicemia virus. Our findings suggest that knowledge of genetic variation of specific, well-characterized, immune-related loci as well as neutral loci in cultured species might be a useful approach when disease prevention and control is a goal. Given that patterns of neutral and immune variation may be decoupled in terms of both variability and response to disease, monitoring of neutral loci alone is unlikely to be informative in regards to important components of immune variation, i.e. overall variation and/or the presence of specific adaptive variants, that may help buffer broodstock from the effects of pathogens.

## Supporting Information

Table S1 Summary statistics for all microsatellites in each sample.
(DOCX)

Data Set S1 Genotypes for all individual in each sample at all microsatellites assayed.
(TXT)
