# Supplementary material for: A Comparison of Neutral and Immune Genetic Variation in Atlantic Salmon, Salmo salar L. in Chilean Aquaculture Facilities
Source: PLoS One. 2014 Jun 11;9(6):e99358. doi: 10.1371/journal.pone.0099358 (PMC4053429; doi:10.1371/journal.pone.0099358)
Supplement: Table S1 — Summary statistics for all microsatellites in each sample. (DOCX) [file pone.0099358.s001.docx]

**Table S1**: Summary statistics for panels of immune-related and neutral microsatellite loci for Atlantic salmon sampled from Chilean aquaculture facilities. Number of samples, n; number of alleles, A; allelic richness, A_R_; gene diversity (expected heterozygosity), H_E_; probability of conformance to the expectations of Hardy-Weinberg equilibrium, P_HW_, year of collection in parenthesis.

| **Immune** |  |  |  |  |  |  |  |
| --- | --- | --- | --- | --- | --- | --- | --- |
|  | **A (2010)** | **B (2010)** | **C (2010)** | **D (2005-2007)** | **E (2006)** | **F (2007)** | **G (2007)** |
| BG9342 |  |  |  |  |  |  |  |
| *n* | 25 | 10 | 12 | 15 | 15 | 13 | 10 |
| A | 14 | 9 | 7 | 11 | 10 | 9 | 6 |
| A_R_ | 9.22 | 8.59 | 6.87 | 9.48 | 7.88 | 8.08 | 5.70 |
| H_E_ | 0.883 | 0.883 | 0.886 | 0.919 | 0.862 | 0.872 | 0.772 |
| P_HW_ | 0.236 | 0.109 | 0.170 | 0.457 | 0.662 | 0.951 | 0.055 |
|  |  |  |  |  |  |  |  |
| *SSa*I001TKU |  |  |  |  |  |  |  |
| *n* | 30 | 11 | 12 | 16 | 16 | 14 | 10 |
| A | 2 | 1 | 1 | 2 | 2 | 2 | 2 |
| A_R_ | 1.95 | 1.00 | 1.00 | 1.97 | 2.00 | 2.00 | 2.00 |
| H_E_ | 0.236 | 0.000 | 0.000 | 0.225 | 0.350 | 0.346 | 0.267 |
| P_HW_ | 0.415 | - | - | 1.000 | 0.543 | 1.000 | 1.000 |
|  |  |  |  |  |  |  |  |
| *SSa*I002TKU |  |  |  |  |  |  |  |
| *n* | 30 | 11 | 12 | 16 | 16 | 14 | 10 |
| A | 5 | 4 | 2 | 4 | 3 | 4 | 3 |
| A_R_ | 3.23 | 3.61 | 1.99 | 3.31 | 2.81 | 3.29 | 2.90 |
| H_E_ | 0.393 | 0.332 | 0.227 | 0.335 | 0.375 | 0.495 | 0.411 |
| P_HW_ | 0.716 | 1.000 | 1.000 | 1.000 | 0.565 | 0.591 | 1.000 |
|  |  |  |  |  |  |  |  |
| *SSa*I003TKU |  |  |  |  |  |  |  |
| *n* | 29 | 11 | 12 | 16 | 16 | 14 | 10 |
| A | 5 | 4 | 5 | 5 | 5 | 6 | 5 |
| A_R_ | 4.42 | 3.79 | 4.49 | 4.31 | 3.69 | 4.81 | 4.80 |
| H_E_ | 0.709 | 0.632 | 0.652 | 0.665 | 0.604 | 0.563 | 0.683 |
| P_HW_ | 0.181 | 0.084 | 0.171 | 0.671 | 1.000 | 0.472 | 0.577 |
|  |  |  |  |  |  |  |  |
| *SSa*I004TKU |  |  |  |  |  |  |  |
| *n* | 30 | 11 | 12 | 16 | 16 | 14 | 10 |
| A | 3 | 2 | 3 | 2 | 3 | 2 | 2 |
| A_R_ | 2.30 | 1.97 | 3.00 | 2.00 | 2.56 | 2.00 | 2.00 |
| H_E_ | 0.491 | 0.173 | 0.644 | 0.513 | 0.435 | 0.511 | 0.389 |
| P_HW_ | 0.171 | 1.000 | 0.837 | 0.348 | 0.274 | 0.589 | 1.000 |
|  |  |  |  |  |  |  |  |
| *SSa*I005TKU |  |  |  |  |  |  |  |
| *n* | 29 | 11 | 12 | 16 | 16 | 14 | 10 |
| A | 2 | 2 | 3 | 3 | 2 | 2 | 3 |
| A_R_ | 2.00 | 2.00 | 2.89 | 2.49 | 2.00 | 1.96 | 2.90 |
| H_E_ | 0.404 | 0.245 | 0.303 | 0.231 | 0.388 | 0.198 | 0.461 |
| P_HW_ | 0.069 | 1.000 | 1.000 | 1.000 | 1.000 | 1.000 | 1.000 |
|  |  |  |  |  |  |  |  |
| *Ssa*I007TKU |  |  |  |  |  |  |  |
| *n* | 30 | 11 | 12 | 16 | 16 | 14 | 10 |
| A | 3 | 2 | 3 | 2 | 3 | 2 | 2 |
| A_R_ | 2.66 | 2.00 | 2.95 | 2.00 | 2.56 | 1.99 | 2.00 |
| H_E_ | 0.436 | 0.245 | 0.572 | 0.492 | 0.458 | 0.258 | 0.533 |
| P_HW_ | 0.562 | 1.000 | 0.213 | 0.159 | 0.188 | 0.218 | 0.246 |
|  |  |  |  |  |  |  |  |
| *SSa*I010TKU |  |  |  |  |  |  |  |
| *n* | 30 | 11 | 12 | 16 | 16 | 14 | 10 |
| A | 4 | 3 | 4 | 3 | 3 | 3 | 2 |
| A_R_ | 3.85 | 2.97 | 3.74 | 2.82 | 2.55 | 2.95 | 1.90 |
| H_E_ | 0.621 | 0.405 | 0.527 | 0.444 | 0.323 | 0.423 | 0.100 |
| P_HW_ | 0.756 | 0.038 | 0.793 | 0.517 | 1.000 | 0.211 | - |
|  |  |  |  |  |  |  |  |
| *SSa*I011TKU |  |  |  |  |  |  |  |
| *n* | 29 | 11 | 12 | 16 | 16 | 14 | 10 |
| A | 3 | 3 | 3 | 3 | 3 | 3 | 3 |
| A_R_ | 2.46 | 3.00 | 2.94 | 2.90 | 2.63 | 2.96 | 2.90 |
| H_E_ | 0.223 | 0.586 | 0.364 | 0.377 | 0.233 | 0.552 | 0.278 |
| P_HW_ | 1.000 | 1.000 | 1.000 | 0.565 | 1.000 | 0.660 | 1.000 |
|  |  |  |  |  |  |  |  |
| *SSa*I012TKU |  |  |  |  |  |  |  |
| *n* | 29 | 11 | 12 | 16 | 16 | 14 | 10 |
| A | 11 | 8 | 10 | 8 | 8 | 7 | 5 |
| A_R_ | 7.96 | 7.40 | 9.01 | 7.33 | 6.61 | 5.97 | 4.80 |
| H_E_ | 0.850 | 0.805 | 0.883 | 0.858 | 0.771 | 0.659 | 0.700 |
| P_HW_ | 0.133 | 0.928 | 0.785 | 0.579 | 0.915 | 0.849 | 0.531 |
|  |  |  |  |  |  |  |  |
| *SSa*I014TKU |  |  |  |  |  |  |  |
| *n* | 28 | 11 | 12 | 16 | 16 | 14 | 10 |
| A | 4 | 3 | 5 | 3 | 3 | 3 | 3 |
| A_R_ | 3.29 | 2.82 | 4.25 | 2.82 | 2.99 | 2.99 | 3.00 |
| H_E_ | 0.591 | 0.323 | 0.587 | 0.496 | 0.638 | 0.629 | 0.628 |
| P_HW_ | 0.451 | 1.000 | 0.580 | 0.227 | 0.644 | 0.431 | 0.017 |
|  |  |  |  |  |  |  |  |
| *SSa*I016TKU |  |  |  |  |  |  |  |
| *n* | 28 | 9 | 12 | 16 | 16 | 14 | 10 |
| A | 5 | 4 | 5 | 4 | 4 | 4 | 3 |
| A_R_ | 4.30 | 4.00 | 4.69 | 3.99 | 4.00 | 3.88 | 3.00 |
| H_E_ | 0.757 | 0.743 | 0.746 | 0.733 | 0.771 | 0.739 | 0.700 |
| P_HW_ | 0.281 | 0.033 | 0.663 | 0.310 | 0.868 | 0.123 | 0.189 |
|  |  |  |  |  |  |  |  |
| MHCII |  |  |  |  |  |  |  |
| *n* | 30 | 11 | 12 | 16 | 16 | 14 | 10 |
| A | 10 | 6 | 7 | 7 | 8 | 5 | 5 |
| A_R_ | 7.15 | 5.61 | 6.19 | 5.91 | 6.38 | 4.76 | 4.99 |
| H_E_ | 0.797 | 0.741 | 0.758 | 0.806 | 0.694 | 0.747 | 0.800 |
| P_HW_ | 0.487 | 0.914 | 0.265 | 0.355 | 0.858 | 0.462 | 0.072 |
|  |  |  |  |  |  |  |  |
| UBA |  |  |  |  |  |  |  |
| *n* | 29 | 11 | 12 | 16 | 16 | 14 | 10 |
| A | 10 | 7 | 9 | 8 | 11 | 10 | 6 |
| A_R_ | 7.20 | 6.61 | 8.36 | 6.32 | 9.22 | 8.34 | 5.60 |
| H_E_ | 0.851 | 0.845 | 0.905 | 0.800 | 0.919 | 0.885 | 0.622 |
| P_HW_ | 0.000 | 0.039 | 0.409 | 0.005 | 0.000 | 0.035 | 0.183 |
|  |  |  |  |  |  |  |  |
| CA769358 |  |  |  |  |  |  |  |
| *n* | 30 | 11 | 12 | 16 | 16 | 14 | 10 |
| A | 8 | 6 | 6 | 6 | 7 | 5 | 4 |
| A_R_ | 6.06 | 5.61 | 5.68 | 5.12 | 5.50 | 4.49 | 4.00 |
| H_E_ | 0.790 | 0.777 | 0.784 | 0.731 | 0.569 | 0.703 | 0.633 |
| P_HW_ | 0.000 | 0.381 | 0.087 | 0.069 | 0.103 | 0.033 | 0.010 |

| **Neutral** |  |  |  |  |  |  |  |
| --- | --- | --- | --- | --- | --- | --- | --- |
|  | **A** | **B** | **C** | **D** | **E** | **F** | **G** |
| *SSsp*1605 |  |  |  |  |  |  |  |
| *n* | 30 | 11 | 12 | 16 | 16 | 14 | 10 |
| A | 9 | 7 | 8 | 7 | 6 | 5 | 6 |
| A_R_ | 5.99 | 6.43 | 7.38 | 5.90 | 5.58 | 4.87 | 5.90 |
| H_E_ | 0.702 | 0.795 | 0.864 | 0.796 | 0.769 | 0.791 | 0.844 |
| P_HW_ | 0.142 | 0.066 | 0.958 | 0.973 | 0.604 | 0.270 | 0.184 |
|  |  |  |  |  |  |  |  |
| *SSsp*2201 |  |  |  |  |  |  |  |
| *n* | 30 | 11 | 12 | 16 | 16 | 14 | 10 |
| A | 13 | 12 | 11 | 14 | 14 | 9 | 7 |
| A_R_ | 8.91 | 10.54 | 9.34 | 10.97 | 10.12 | 7.41 | 6.89 |
| H_E_ | 0.877 | 0.918 | 0.883 | 0.933 | 0.892 | 0.841 | 0.856 |
| P_HW_ | 0.825 | 0.134 | 0.515 | 0.303 | 0.731 | 0.146 | 0.846 |
|  |  |  |  |  |  |  |  |
| *SSsp*2210 |  |  |  |  |  |  |  |
| *n* | 30 | 11 | 12 | 16 | 14 | 10 | 30 |
| A | 9 | 9 | 9 | 7 | 6 | 6 | 5 |
| A_R_ | 6.90 | 7.91 | 8.12 | 6.16 | 5.66 | 5.55 | 4.80 |
| H_E_ | 0.844 | 0.841 | 0.871 | 0.815 | 0.777 | 0.747 | 0.767 |
| P_HW_ | 0.936 | 0.313 | 0.663 | 0.426 | 0.841 | 0.531 | 1.000 |
|  |  |  |  |  |  |  |  |
| *SSsp*2213 |  |  |  |  |  |  |  |
| *n* | 30 | 11 | 12 | 16 | 16 | 14 | 10 |
| A | 8 | 7 | 6 | 10 | 9 | 7 | 5 |
| A_R_ | 6.47 | 6.61 | 5.48 | 8.03 | 7.70 | 6.40 | 4.90 |
| H_E_ | 0.825 | 0.845 | 0.773 | 0.860 | 0.833 | 0.846 | 0.778 |
| P_HW_ | 0.735 | 0.156 | 0.071 | 0.017 | 0.127 | 0.746 | 0.834 |
|  |  |  |  |  |  |  |  |
| *SSsp*2215 |  |  |  |  |  |  |  |
| *n* | 30 | 11 | 12 | 16 | 16 | 14 | 10 |
| A | 12 | 12 | 9 | 9 | 10 | 6 | 6 |
| A_R_ | 8.51 | 10.67 | 7.93 | 7.05 | 7.91 | 5.40 | 5.90 |
| H_E_ | 0.877 | 0.918 | 0.860 | 0.846 | 0.865 | 0.788 | 0.811 |
| P_HW_ | 0.531 | 0.835 | 0.031 | 0.407 | 0.333 | 0.132 | 0.369 |
|  |  |  |  |  |  |  |  |
| *SSsp*2216 |  |  |  |  |  |  |  |
| *n* | 30 | 11 | 12 | 16 | 16 | 14 | 10 |
| A | 11 | 10 | 8 | 11 | 11 | 6 | 6 |
| A_R_ | 7.66 | 9.04 | 6.99 | 8.18 | 8.59 | 5.57 | 5.80 |
| H_E_ | 0.845 | 0.891 | 0.833 | 0.813 | 0.863 | 0.791 | 0.778 |
| P_HW_ | 0.277 | 0.370 | 0.286 | 0.602 | 0.669 | 0.712 | 0.215 |
|  |  |  |  |  |  |  |  |
| *SSsp*G7 |  |  |  |  |  |  |  |
| *n* | 30 | 11 | 12 | 16 | 16 | 14 | 10 |
| A | 14 | 7 | 8 | 9 | 10 | 8 | 5 |
| A_R_ | 9.14 | 6.61 | 7.57 | 7.29 | 7.88 | 6.33 | 4.80 |
| H_E_ | 0.902 | 0.855 | 0.871 | 0.860 | 0.840 | 0.753 | 0.611 |
| P_HW_ | 0.005 | 0.231 | 0.920 | 0.184 | 0.522 | 1.000 | 0.695 |
|  |  |  |  |  |  |  |  |
| *Ssa*14 |  |  |  |  |  |  |  |
| *n* | 30 | 11 | 12 | 16 | 14 | 10 | 30 |
| A | 2 | 2 | 2 | 2 | 2 | 2 | 2 |
| A_R_ | 2.00 | 2.00 | 2.00 | 2.00 | 2.00 | 2.00 | 2.00 |
| H_E_ | 0.472 | 0.245 | 0.515 | 0.517 | 0.446 | 0.352 | 0.267 |
| P_HW_ | 1.000 | 1.000 | 0.289 | 1.000 | 0.591 | 0.490 | 1.000 |
|  |  |  |  |  |  |  |  |
| *Ssa*85 |  |  |  |  |  |  |  |
| *n* | 30 | 11 | 12 | 16 | 16 | 14 | 10 |
| A | 15 | 9 | 8 | 8 | 11 | 6 | 10 |
| A_R_ | 9.79 | 8.53 | 7.34 | 7.19 | 7.98 | 5.86 | 9.49 |
| H_E_ | 0.911 | 0.900 | 0.852 | 0.846 | 0.835 | 0.841 | 0.900 |
| P_HW_ | 0.120 | 0.941 | 0.853 | 0.314 | 0.949 | 0.232 | 0.054 |
|  |  |  |  |  |  |  |  |
| *Ssa*197 |  |  |  |  |  |  |  |
| *n* | 30 | 11 | 12 | 16 | 16 | 14 | 10 |
| A | 14 | 11 | 8 | 10 | 10 | 6 | 7 |
| A_R_ | 9.02 | 10.01 | 7.09 | 8.56 | 8.63 | 4.93 | 6.69 |
| H_E_ | 0.893 | 0.918 | 0.799 | 0.902 | 0.902 | 0.736 | 0.794 |
| P_HW_ | 0.327 | 1.000 | 0.088 | 0.781 | 0.273 | 0.479 | 0.102 |
|  |  |  |  |  |  |  |  |
| *Ssa*171 |  |  |  |  |  |  |  |
| *n* | 30 | 11 | 12 | 16 | 16 | 14 | 10 |
| A | 10 | 12 | 9 | 9 | 9 | 7 | 7 |
| A_R_ | 7.82 | 10.49 | 7.87 | 7.44 | 7.47 | 6.35 | 6.88 |
| H_E_ | 0.882 | 0.905 | 0.799 | 0.865 | 0.840 | 0.786 | 0.839 |
| P_HW_ | 0.115 | 0.926 | 0.377 | 0.556 | 0.933 | 0.059 | 0.159 |
|  |  |  |  |  |  |  |  |
| *Ssa*202 |  |  |  |  |  |  |  |
| *n* | 30 | 11 | 12 | 16 | 16 | 14 | 10 |
| A | 9 | 8 | 12 | 8 | 7 | 6 | 6 |
| A_R_ | 6.38 | 7.27 | 10.37 | 5.98 | 5.06 | 5.45 | 5.80 |
| H_E_ | 0.816 | 0.845 | 0.924 | 0.658 | 0.585 | 0.723 | 0.778 |
| P_HW_ | 0.8842 | 0.5089 | 0.8455 | 0.5151 | 0.4428 | 0.1423 | 0.9001 |
|  |  |  |  |  |  |  |  |
| *Ss0*SL85 |  |  |  |  |  |  |  |
| *n* | 30 | 11 | 12 | 16 | 16 | 14 | 10 |
| A | 9 | 8 | 9 | 6 | 5 | 7 | 5 |
| A_R_ | 6.85 | 7.40 | 7.89 | 5.48 | 4.54 | 6.05 | 5.00 |
| H_E_ | 0.794 | 0.859 | 0.860 | 0.817 | 0.717 | 0.802 | 0.828 |
| P_HW_ | 0.595 | 0.738 | 0.796 | 0.528 | 0.474 | 0.004 | 0.080 |
|  |  |  |  |  |  |  |  |
| *Ss0*SL311 |  |  |  |  |  |  |  |
| *n* | 28 | 10 | 12 | 16 | 16 | 14 | 9 |
| A | 14 | 14 | 10 | 13 | 12 | 9 | 8 |
| A_R_ | 9.13 | 13.08 | 8.43 | 9.85 | 9.31 | 7.83 | 8.00 |
| H_E_ | 0.896 | 0.961 | 0.826 | 0.915 | 0.904 | 0.887 | 0.847 |
| P_HW_ | 0.679 | 1.000 | 0.684 | 0.311 | 0.930 | 0.238 | 0.191 |
|  |  |  |  |  |  |  |  |
| *Ss0*SL438 |  |  |  |  |  |  |  |
| *n* | 30 | 11 | 12 | 16 | 16 | 14 | 10 |
| A | 10 | 9 | 7 | 8 | 7 | 6 | 3 |
| A_R_ | 6.52 | 8.55 | 6.24 | 5.80 | 5.67 | 5.27 | 3.00 |
| H_E_ | 0.810 | 0.909 | 0.811 | 0.744 | 0.810 | 0.750 | 0.494 |
| P_HW_ | 0.845 | 0.280 | 0.063 | 0.035 | 0.098 | 0.949 | 0.133 |
